# An Antidote to Chloral

**Published:** 1870-06

**Authors:** 


### An Antidote to Chloral.

M. Liebreich, of Berlin, after having studied and made known the physiological properties of hydrate of chloral, has directed his attention to the obtainment of a remedy which may counteract the baneful effects of the substance. This antidote he asserts to be strychnia. The results of M. Liebreich's novel researches were laid before the French Academy of Sciences a few days ago, by Professor Wurtz, of the Paris Faculty. M. Wurtz stated that the attention of the Berlin experimenter had been drawn to strychnia by the observation of a case of trismus, which, after having persisted eight days, was immediately cured on the employment of chloral. This led him to produce, artificially, the phenomena of tetanus in animals by the administration of strychnia, in order to study the effects of chloral on this artificial condition. He then observed that chloral diminished the effects of strychnia, provided it were exhibited very soon after the administration of the alkaloid.

Another important result was obtained when M. Liebreich, pursuing his researches, discovered the effect of strychnine on animals poisoned by large doses of chloral.

He then made out that strychnia, administered after too heavy a dose of chloral, shortens and destroys the effects of this substance, yet without producing the injurious action which it exhibits in ordinary cases. Therefore, M. Liebreich proposes to employ injections of nitrate of strychnia as an antidote in cases where accidents have occurred through an overdose of chloral or chloroform.—*Lancet.*

